# Renal cancer and Wegener's granulomatosis: a case report

**DOI:** 10.1186/1477-7819-9-165

**Published:** 2011-12-13

**Authors:** Uros Bumbasirevic, Dejan Dragicevic, Aleksandar Janicic, Vesna Cemerikic-Martinovic, Milica Cekerevac, Vuk Aleksic, Cane Tulic

**Affiliations:** 1 Clinical Center of Serbia, Clinic of Urology, Resavska 51, 11000 Belgrade, Serbia; 2 Faculty of Medicine, University of Belgrade, Dr. Subotica 8, Belgrade, Serbia; 3 Beo-Lab, Resavska 60, Belgrade, Serbia

**Keywords:** Wegener's granulomatosis, Renal Cell Carcinoma, Cyclophosphamide, Renal mass

## Abstract

Wegener's granulomatosis (WG) is a systemic disorder characterized by necrotizing vasculitis involving the respiratory tract, and in most cases, the kidneys. The most common manifestation of WG in the kidneys is segmental necrotizing glomerulonephritis. The presence of a renal mass as a manifestation of WG is rare. We report a patient with WG in whom a CT scan revealed an infiltrating mass in the lower portion of the left kidney. After surgical exploration, we performed an open radical nephrectomy. Histopathology showed clear cell type renal cell carcinoma (RCC). RCC associated with WG has been reported in only a few cases, and in most of them, the diseases started simultaneously, suggesting common pathogenetic pathways. Long-term immunosuppressive treatment is a known risk factor in the development of malignancies, so occurrence of RCC in WG has been proposed as a side effect of cyclophosphamide treatment. Furthermore, it is important to make a differential diagnosis between RCC and pseudotumors in WG as they cannot be distinguished solely on basis of imaging findings. Due to the higher risk of urologic malignancies, more frequent checkups and screening of WG patients should be considered.

## Background

Wegener's granulomatosis (WG) is a disease of unknown etiology characterized by systemic necrotizing granulomatous vasculitis, which primarily involves the upper respiratory tract and the lungs. Although renal involvement is present in only 20% of patients at time of diagnosis, it eventually occurs in approximately 80% of all patients suffering from WG. The most common manifestation of WG in the kidney is segmental necrotizing glomerulonephritis with proteinuria that often leads to rapidly progressive renal failure [1]. The presence of a renal mass in patients with WG is rare; only a few cases have been reported [2-7]. Pathologic analysis of the masses in these cases revealed them to be pseudotumors or renal cell carcinoma (RCC). In most cases reporting WG and RCC, the diseases developed simultaneously.

Cyclophosphamide is a mainstay therapy for WG [8]. There is up to 33-fold increased risk for development of urinary bladder carcinoma, and the risk is cumulative dose dependent and related to the duration of therapy with cyclophosphamide [9]. However, RCC development with cyclophosphamide therapy has been reported in only few cases and a direct connection is yet to be determined [3,4].

We report a case in which renal cancer developed thirteen years after WG was diagnosed.

## Case Presentation

Our patient is a 55-year-old man. His symptoms started in November 1996 with a high fever and cough. About ten days later, he developed joint, back, and chest pains, epistaxis, hemoptysis, dyspnea, and a vesicular hemorrhagic rash. He lost 15 kg in one month upon which, he was hospitalized.

The patient presented with a high fever, blood pressure at 135/110 mmHg, hemoglobin at 14 g/dl, an erythrocyte sedimentation rate of 80 mm/h, white blood cell count of 7,5 × 10^9^/L, and platelet count of 200 × 10^9^/L. A 24-hour urinary protein excretion was measured at 0.9 g/L with microscopic hematuria, and he had a positive serum test for antineutrophil cytoplasmic antibodies (ANCA). A chest x-ray showed multiple nodular and patchy shadows throughout both lungs and a prominent left hilum. He was diagnosed with WG and started with pulse doses of glucocorticosteroids (IV) after which cyclophosphamide (IV) was instituted, with tapering of corticosteroid dose. The symptoms soon resolved and complete remission was achieved.

In the second year of his ongoing treatment, because of the development of diabetes mellitus, steroid treatment was stopped, and the therapy was continued with cyclophosphamide *per os *only. The patient experienced two periods of relapse, and after reinstituting therapy (three pulses of methylprednisolone 500 mg i.v. and then two pulses of cyclophosphamide 1000 mg, after two weeks oral cyclophosphamide 100 mg/d therapy was reinstituted), went into remission. The total cumulative dose of cyclophosphamide was about 150 grams.

**Figure 1:**
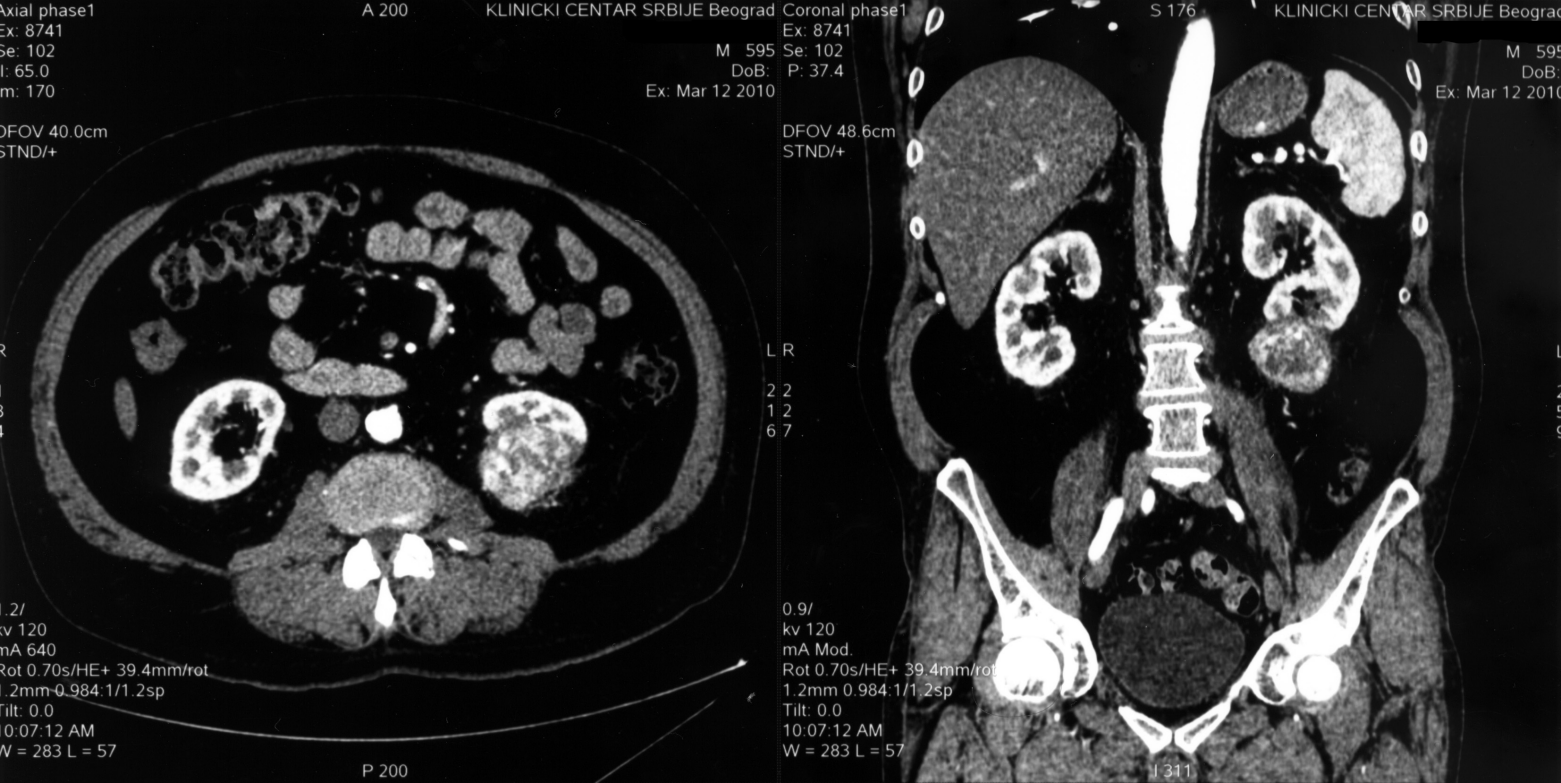
**Abdominal CT scan revealing an infiltrating mass in lower portion of the left kidney**.

**Figure 2:**
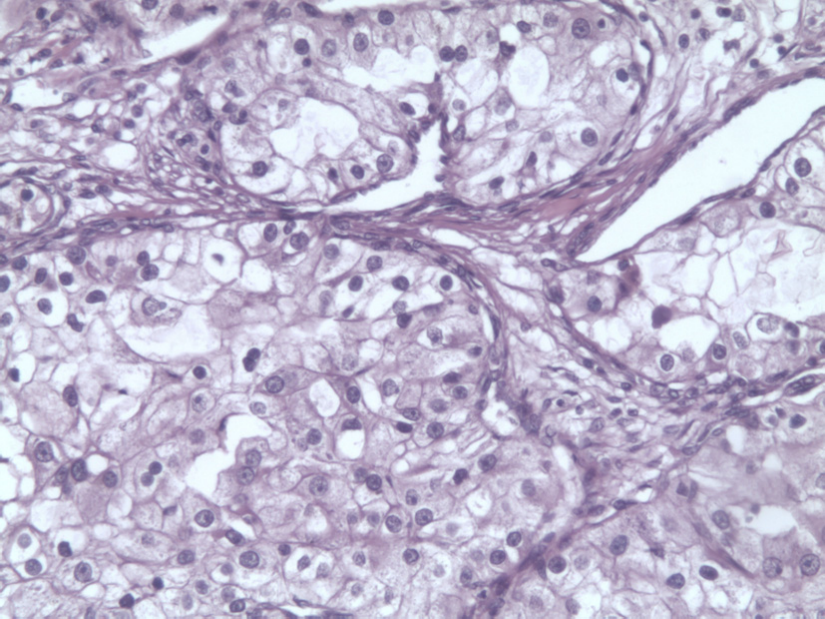
**Light photomicrograph showing clear cell type renal cell carcinoma (RCC)**. H/E staining, 200 × magnification.

**Figure 3:**
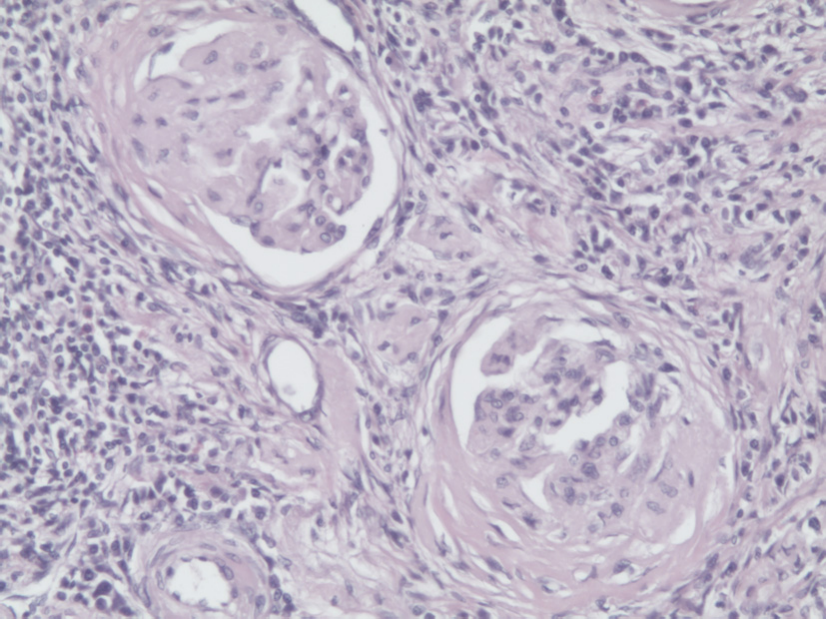
**Non-neoplastic kidney parenchyma with signs of glomerular sclerosis and vessels showing fibromuscular hyperplasia**. H/E staining, 200 × magnification.

Thirteen years later, the patient was admitted to our hospital due to pain in the left lumbar region, which began two months earlier. He is a nonsmoker; his body mass index was 26; with no exposure to occupational carcinogens, and a negative familial history for renal malignancies. A CT scan revealed an infiltrating mass, 53 mm at its widest diameter, in the lower portion of the left kidney (Figure 1). Laboratory analysis of blood and urine showed normal values while the ANCA test was negative, suggesting that his WG was still in remission. After surgical exploration, we performed an open radical nephrectomy, and a histological examination revealed a clear cell type renal cell carcinoma, stage pT3a, grade 2 (Figure 2). In the non-neoplastic kidney parenchyma there were signs of glomerular sclerosis and vessels showing fibromuscular hyperplasia (Figure 3). He is being followed up on regularly and is in remission of WG and RCC.

## Discussion

In 1999, Tatsis et al. [2] reported significant association between RCC and WG, and suggested that malignancy is, in some cases, a trigger for the development of WG, e.g. cytokine production by malignant cells may participate in the etiology of autoimmunity and vasculitis. In our patient, RCC developed during the remission of WG which was diagnosed thirteen years earlier.

Immunosuppressive therapy, primarily cyclophosphamide, and steroids have markedly improved survival and remission rates in WG patients [8]. However, this therapy is associated with a significantly increased risk of urinary bladder cancer development. The risk is directly related to the duration of therapy and the total dosage [9]. In 2009, Deger et al. [3] reported RCC after eight years of WG treatment with cyclophosphamide and azathioprine, proposing the possible connection between immunosuppressive therapy and renal cancer. In 1996, Odeh [4] reported for the first time, the development of RCC after treatment with cyclophosphamide in a WG patient. Our case may also suggest the association of immunosuppressive therapy with the development of malignant tumors of the kidney. There were no other known risk factors for renal cancer in our patient. Nevertheless, RCC may have developed incidentally. Further studies are needed to reveal a definitive connection between WG and RCC as well as the possible influence of long-term immunosuppressive therapy on the development of RCC.

In addition, in patients with WG and a renal mass, there is a differential diagnostic problem. Frigui et al. [5] reported a review of literature documenting thirteen cases of renal masses, pathologically verified pseudotumors, as a consequence of WG itself. However, differential diagnosis between RCC and pseudotumors cannot be made solely on the basis of imaging findings. Villa-Forte et al. [10] reported a case of simultaneous development of RCC and WG renal lesions and proposed renal biopsy to confirm the diagnosis of suspected WG and repetition of imaging studies to determine the resolution of mass lesions after appropriate treatment.

## Conclusion

In conclusion, we suggest that in WG patients on immunosuppressive therapy in whom the disease is in a documented remission, renal masses should be presumed to be malignant, generally RCC, but carcinoma of the renal pelvis should also be kept in mind when dealing with patients with a cyclophosphamide therapy. Definitive diagnosis can only be made by renal biopsy.

Due to the higher risk of urologic malignancies, more frequent checkups and screening of WG patients should also be considered.

## Consent

Written informed consent was obtained from the patient for publication of this Case report and any accompanying images. A copy of the written consent is available for review by the Editor-in-Chief of this journal.

## List of abbreviations

WG: Wegener's granulomatosis; RCC: Renal Cell Carcinoma; ANCA: Antineutrophil Cytoplasmic Antibodies.

## Competing interests

The authors declare that they have no competing interests.

## Authors' contributions

UB prepared the manuscript and the literature search; CT, DD, AJ and UB did the surgery; CT and DD reviewed and edited the manuscript; VA collected the data; VCM and MC did the pathologic analysis.

All authors read and approved the final manuscript.
